# (1*Z*,2*E*)-1-(3,4-Diphenyl-2,3-di­hydro-1,3-thia­zol-2-yl­idene)-2-(1-*p*-tolyl­ethyl­idene)hydrazine

**DOI:** 10.1107/S1600536813020254

**Published:** 2013-07-27

**Authors:** Shaaban K. Mohamed, Joel T. Mague, Mehmet Akkurt, Alaa A. Hassan, Mustafa R. Albayati

**Affiliations:** a Chemistry and Environmental Division, Manchester Metropolitan University, Manchester M1 5GD, England; b Chemistry Department, Faculty of Science, Mini University, 61519 El-Minia, Egypt; c Department of Chemistry, Tulane University, New Orleans, LA 70118, USA; d Department of Physics, Faculty of Sciences, Erciyes University, 38039 Kayseri, Turkey; e Kirkuk University, College of Science, Department of Chemistry, Kirkuk, Iraq

## Abstract

In the title compound, C_24_H_21_N_3_S, the thia­zole ring makes dihedral angles of 52.03 (6), 62.63 (6) and 12.35 (6)°, respectively, with the two phenyl rings and the benzene ring. In the crystal, weak C—H⋯π inter­actions occur between inversion-related mol­ecules.

## Related literature

For the syntheses and bioactivity of thia­zole-containing compounds, see: Siddiqui *et al.* (2009 ▶); Ramla *et al.* (2006 ▶); Popsavin *et al.* (2007 ▶); Kumar *et al.* (2007 ▶); Pandeya *et al.* (1999 ▶); Narayana *et al.* (2004 ▶); Shiradkar *et al.* (2007 ▶); Amin *et al.* (2008 ▶); Shih & Ying (2004 ▶); Andreani *et al.* (1987 ▶).
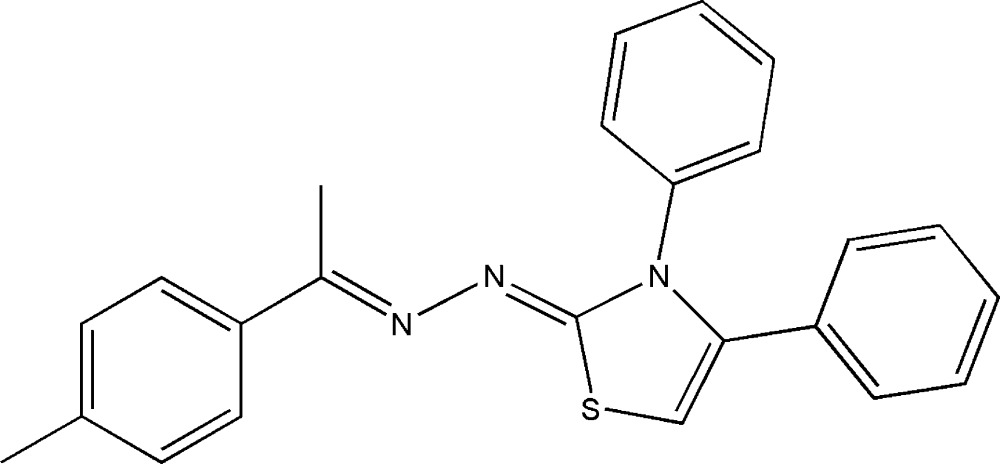


## Experimental

### 

#### Crystal data

C_24_H_21_N_3_S
*M* *_r_* = 383.51
Triclinic, 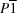

*a* = 7.9370 (8) Å
*b* = 10.7587 (11) Å
*c* = 11.9325 (13) Å
α = 94.922 (2)°
β = 97.436 (2)°
γ = 95.383 (2)°
*V* = 1000.95 (18) Å^3^
*Z* = 2
Mo *K*α radiation
μ = 0.18 mm^−1^
*T* = 150 K
0.30 × 0.15 × 0.12 mm

#### Data collection

Bruker SMART APEX CCD diffractometer
Absorption correction: multi-scan (*SADABS*; Bruker, 2013 ▶) *T*_min_ = 0.83, *T*_max_ = 0.98
17908 measured reflections
4870 independent reflections
4287 reflections with *I* > 2σ(*I*)
*R*_int_ = 0.034

#### Refinement

*R*[*F* ^2^ > 2σ(*F* ^2^)] = 0.043
*wR*(*F* ^2^) = 0.119
*S* = 1.06
4870 reflections
255 parameters
H-atom parameters constrained
Δρ_max_ = 0.33 e Å^−3^
Δρ_min_ = −0.30 e Å^−3^

### 

Data collection: *APEX2* (Bruker, 2013 ▶); cell refinement: *SAINT* (Bruker, 2013 ▶); data reduction: *SAINT*; program(s) used to solve structure: *SHELXS97* (Sheldrick, 2008 ▶); program(s) used to refine structure: *SHELXL97* (Sheldrick, 2008 ▶); molecular graphics: *ORTEP-3 for Windows* (Farrugia, 2012 ▶) and *DIAMOND* (Brandenburg & Putz, 2012 ▶); software used to prepare material for publication: *WinGX* (Farrugia, 2012 ▶) and *PLATON* (Spek, 2009 ▶).

## Supplementary Material

Crystal structure: contains datablock(s) global, I. DOI: 10.1107/S1600536813020254/xu5722sup1.cif

Structure factors: contains datablock(s) I. DOI: 10.1107/S1600536813020254/xu5722Isup2.hkl

Supplementary material file. DOI: 10.1107/S1600536813020254/xu5722Isup3.cml

Additional supplementary materials:  crystallographic information; 3D view; checkCIF report

## Figures and Tables

**Table 1:** Hydrogen-bond geometry (Å, °) *Cg*4 is the centroid of the C18–C23 ring.

*D*—H⋯*A*	*D*—H	H⋯*A*	*D*⋯*A*	*D*—H⋯*A*
C15—H15⋯*Cg*4^i^	0.95	2.71	3.6170 (16)	160
C16—H16*A*⋯*Cg*4^ii^	0.98	2.77	3.5984 (16)	143

Symmetry codes: (i) 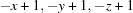; (ii) 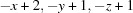.

**Fig. 1.**
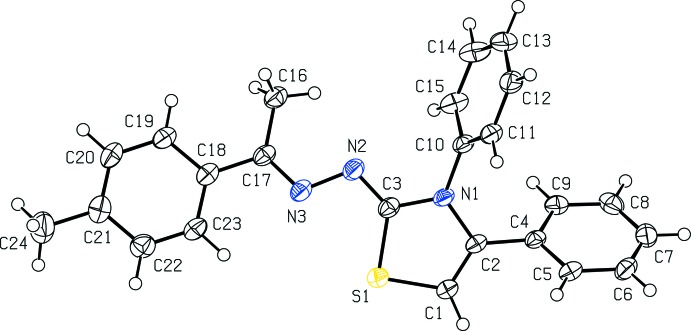
Perspective view of the title compound with 50% probability displacement ellipsoids.

**Fig. 2.**
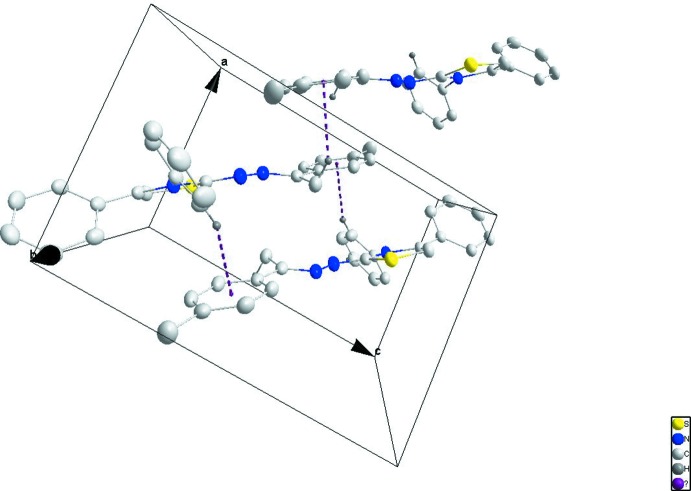
Closeup view (down b) of the C—H···π interactions.

## supplementary crystallographic information

### Comment

1,3-Thiazole containing compounds are a great class of sulfur heterocyclic molecules due to their vital interest in pharmaceutical and industrial chemistry. Numerous of synthesized active thiazole scaffold molecules having diverse of biologically activities have been reported (Ramla *et al.*, 2006; Popsavin *et al.*, 2007; Kumar *et al.*, 2007; Pandeya *et al*., 1999; Narayana *et al.*, 2004; Shiradkar *et al.*, 2007; Amin *et al.*, 2008; Shih & Ying, 2004; Andreani *et al.*, 1987). In addition, thiazole compounds have not only found in natural molecules such as Thiamin (vitamin B1) but also have been found in many drugs such as Abafungin (antifungal drug), Bleomycine and Tiazofurin (antineoplastic drug), Ritonavir (antiretroviral drug) and Sulfathiazol (antimicrobial drug) (Siddiqui *et al.*, 2009).

In the title compound (I), (Fig. 1), the (S1/N1/C1–C3) thiazole ring is essentially planar with a maximum deviation of -0.006 (1) Å for C2. The dihedral angles between the thiazole ring, and the phenyl rings (C4–C9 and C10–C15) and the benzene ring (C18—C23) are 52.03 (6), 62.63 (6) and 12.35 (6)°, respectively.

In the crystal structure, the title molecules pack in a layer structure with the layers approximately parallel to [101] and held together by a combination of C—H···S hydrogen bonds (Table 1 and Fig. 2) and C—H···π interactions (Fig. 3: H15···*Cg*1 = 2.71 Å; C15—H15···*Cg*1 = 160° and H16*a*···*Cg*2 = 2.77 Å; C16—H16*a*···*Cg*2 = 143° where *Cg*1 is the centroid of the ring C18–C23 at 1 - *x*, 1 - *y*, 1 - *z* and *Cg*2 is the centroid of the ring C18–C23 at 2 - *x*, 1 - *y*, 1 - *z*).

### Experimental

A solution of 283 mg (1 mmol) (2E)-2-[1-(4-methylphenyl)ethylidene]-*N*-phenylhydrazinecarbothioamide in 15 ml e thanol was added dropwise to a solution of 199 mg (1 mmol) 2-bromo-1-phenylethanone in 20 ml e thanol and few drops of piperidine. The reaction mixture was stirred and refluxed for 6 h. On cooling the solid product was precipitated, filtered off and recrystallized from ethanol to furnish translucent yellow blocks (*M*.p. 509 - 511 K) suitable for X-ray diffraction.

IR [ν, cm^-1^,KBr]: 3052 (Ar—CH), 2941, 2863 (Ali – CH), 1594 (Ar—C=C), 1350(C=S); ^1^H– NMR [δ, p.p.m., CDCl_3_]: 1.26, 1.65, 1.94 and 2.20 (10H, cyclohexane – CH_2_), 7.707,7.51, 7.55 (SH, Ar –H); ^13^C–NMR [δ, p.p.m., CDCl_3_]: 23.58, 24.52 and 33.90 (cyclohexane- CH_2_), 112.19 (spiro Cx b), 127.81, 129.87, 130.22 (Ar- CH), 135.07 (Ar-c), 187.83 (C=S).

### Refinement

H atoms were placed in geometrically idealized positions and constrained to ride on their parent atoms with C—H = 0.95 (aromatic H) and 0.98 Å (methyl H), with *U*_iso_(H) = 1.2 *U*_iso_(C) for aromatic H atoms and *U*_iso_(H) = 1.5 *U*_iso_(C) for methyl H atoms.

### Crystal data

**Table:**

C_24_H_21_N_3_S	*Z* = 2
*M**_r_* = 383.51	*F*(000) = 404
Triclinic, *P*1	*D*_x_ = 1.273 Mg m^−^^3^
Hall symbol: -P 1	Mo *K*α radiation, λ = 0.71073 Å
*a* = 7.9370 (8) Å	Cell parameters from 9946 reflections
*b* = 10.7587 (11) Å	θ = 2.5–29.1°
*c* = 11.9325 (13) Å	µ = 0.18 mm^−^^1^
α = 94.922 (2)°	*T* = 150 K
β = 97.436 (2)°	Block, translucent yellow
γ = 95.383 (2)°	0.30 × 0.15 × 0.12 mm
*V* = 1000.95 (18) Å^3^	

### Data collection

**Table:**

Bruker SMART APEX CCD diffractometer	4870 independent reflections
Radiation source: fine-focus sealed tube	4287 reflections with *I* > 2σ(*I*)
Graphite monochromator	*R*_int_ = 0.034
φ and ω scans	θ_max_ = 28.2°, θ_min_ = 2.5°
Absorption correction: multi-scan (*SADABS*; Bruker, 2013)	*h* = −10→10
*T*_min_ = 0.83, *T*_max_ = 0.98	*k* = −14→13
17908 measured reflections	*l* = −15→15

### Refinement

**Table:**

Refinement on *F*^2^	0 restraints
Least-squares matrix: full	Hydrogen site location: inferred from neighbouring sites
*R*[*F*^2^ > 2σ(*F*^2^)] = 0.043	H-atom parameters constrained
*wR*(*F*^2^) = 0.119	W = 1/[Σ^2^(*F*O^2^) + (0.0663*P*)^2^ + 0.2395*P*] WHERE *P* = (*F*O^2^ + 2*F*C^2^)/3
*S* = 1.06	(Δ/σ)_max_ < 0.001
4870 reflections	Δρ_max_ = 0.33 e Å^−^^3^
255 parameters	Δρ_min_ = −0.30 e Å^−^^3^

### Special details

**Table:**

Experimental. The diffraction data were obtained from 3 sets of 400 frames, each of width 0.5° in ω, colllected at φ = 0.00, 90.00 and 180.00° and 2 sets of 800 frames, each of width 0.45° in φ, collected at ω = -30.00 and 210.00°. The scan time was 20 sec/frame.
Geometry. Bond distances, angles *etc*. have been calculated using the rounded fractional coordinates. All su's are estimated from the variances of the (full) variance-covariance matrix. The cell e.s.d.'s are taken into account in the estimation of distances, angles and torsion angles
Refinement. Refinement on *F*^2^ for ALL reflections except those flagged by the user for potential systematic errors. Weighted *R*-factors *wR* and all goodnesses of fit *S* are based on *F*^2^, conventional *R*-factors *R* are based on *F*, with *F* set to zero for negative *F*^2^. The observed criterion of *F*^2^ > σ(*F*^2^) is used only for calculating -*R*-factor-obs *etc*. and is not relevant to the choice of reflections for refinement. *R*-factors based on *F*^2^ are statistically about twice as large as those based on *F*, and *R*-factors based on ALL data will be even larger.

### Fractional atomic coordinates and isotropic or equivalent isotropic displacement parameters (Å^2^)

**Table:**

	*x*	*y*	*z*	*U*_iso_*/*U*_eq_	
S1	0.39566 (4)	0.41513 (3)	0.16060 (3)	0.0296 (1)	
N1	0.40562 (13)	0.65478 (10)	0.15561 (9)	0.0266 (3)	
N2	0.58080 (15)	0.59963 (11)	0.31261 (10)	0.0320 (3)	
N3	0.63650 (14)	0.49413 (11)	0.36084 (9)	0.0304 (3)	
C1	0.27238 (16)	0.47641 (12)	0.05087 (11)	0.0294 (3)	
C2	0.28971 (15)	0.60194 (12)	0.06040 (10)	0.0250 (3)	
C3	0.47515 (15)	0.56729 (12)	0.22097 (10)	0.0269 (3)	
C4	0.18436 (15)	0.68175 (12)	−0.00790 (10)	0.0250 (3)	
C5	0.16266 (16)	0.66568 (13)	−0.12623 (11)	0.0299 (4)	
C6	0.05372 (18)	0.73637 (14)	−0.18849 (12)	0.0353 (4)	
C7	−0.03595 (18)	0.82156 (13)	−0.13387 (13)	0.0371 (4)	
C8	−0.01560 (18)	0.83778 (13)	−0.01614 (13)	0.0354 (4)	
C9	0.09520 (17)	0.76964 (12)	0.04655 (11)	0.0298 (3)	
C10	0.46544 (15)	0.78546 (12)	0.17887 (11)	0.0268 (3)	
C11	0.54876 (16)	0.84599 (12)	0.10073 (11)	0.0296 (4)	
C12	0.60307 (18)	0.97346 (14)	0.12303 (12)	0.0357 (4)	
C13	0.5727 (2)	1.03903 (14)	0.22148 (14)	0.0417 (5)	
C14	0.4910 (2)	0.97690 (16)	0.30020 (14)	0.0445 (5)	
C15	0.43855 (18)	0.84986 (14)	0.27966 (12)	0.0361 (4)	
C16	0.72732 (18)	0.64349 (13)	0.53188 (11)	0.0338 (4)	
C17	0.70933 (16)	0.51734 (13)	0.46504 (11)	0.0281 (3)	
C18	0.78315 (15)	0.41138 (13)	0.51776 (10)	0.0283 (4)	
C19	0.89132 (17)	0.43040 (14)	0.62146 (11)	0.0323 (4)	
C20	0.97073 (17)	0.33273 (15)	0.66605 (11)	0.0347 (4)	
C21	0.94400 (18)	0.21266 (15)	0.61090 (12)	0.0357 (4)	
C22	0.83119 (19)	0.19244 (15)	0.50894 (12)	0.0371 (4)	
C23	0.75304 (17)	0.28937 (14)	0.46339 (11)	0.0328 (4)	
C24	1.0340 (3)	0.10791 (18)	0.65903 (15)	0.0536 (6)	
H1	0.20060	0.42600	−0.00920	0.0350*	
H5	0.22240	0.60630	−0.16440	0.0360*	
H6	0.04080	0.72610	−0.26910	0.0420*	
H7	−0.11130	0.86890	−0.17690	0.0440*	
H8	−0.07780	0.89580	0.02150	0.0430*	
H9	0.11080	0.78260	0.12710	0.0360*	
H11	0.56870	0.80090	0.03250	0.0360*	
H12	0.66140	1.01550	0.07020	0.0430*	
H13	0.60740	1.12650	0.23550	0.0500*	
H14	0.47120	1.02200	0.36840	0.0530*	
H15	0.38460	0.80710	0.33410	0.0430*	
H16A	0.84650	0.68030	0.54040	0.0510*	
H16B	0.69340	0.63410	0.60710	0.0510*	
H16C	0.65380	0.69850	0.49190	0.0510*	
H19	0.91060	0.51160	0.66190	0.0390*	
H20	1.04510	0.34850	0.73600	0.0420*	
H22	0.80820	0.11030	0.47050	0.0440*	
H23	0.67750	0.27310	0.39400	0.0390*	
H24A	1.14520	0.10640	0.63190	0.0800*	
H24B	0.96450	0.02770	0.63440	0.0800*	
H24C	1.05080	0.12150	0.74220	0.0800*	

### Atomic displacement parameters (Å^2^)

**Table:**

	*U*^11^	*U*^22^	*U*^33^	*U*^12^	*U*^13^	*U*^23^
S1	0.0294 (2)	0.0278 (2)	0.0296 (2)	−0.0005 (1)	−0.0006 (1)	0.0023 (1)
N1	0.0260 (5)	0.0270 (5)	0.0233 (5)	−0.0051 (4)	−0.0028 (4)	0.0006 (4)
N2	0.0311 (5)	0.0341 (6)	0.0279 (5)	−0.0028 (4)	−0.0036 (4)	0.0043 (4)
N3	0.0277 (5)	0.0358 (6)	0.0255 (5)	−0.0031 (4)	−0.0008 (4)	0.0040 (4)
C1	0.0282 (6)	0.0294 (6)	0.0273 (6)	−0.0009 (5)	−0.0031 (5)	−0.0007 (5)
C2	0.0221 (5)	0.0291 (6)	0.0213 (5)	−0.0028 (4)	0.0002 (4)	−0.0010 (4)
C3	0.0238 (5)	0.0312 (6)	0.0243 (6)	−0.0028 (5)	0.0020 (4)	0.0027 (5)
C4	0.0213 (5)	0.0258 (6)	0.0253 (6)	−0.0045 (4)	−0.0002 (4)	0.0004 (4)
C5	0.0262 (6)	0.0337 (7)	0.0265 (6)	−0.0027 (5)	−0.0002 (5)	−0.0026 (5)
C6	0.0334 (7)	0.0401 (8)	0.0277 (6)	−0.0067 (6)	−0.0064 (5)	0.0051 (5)
C7	0.0299 (6)	0.0306 (7)	0.0480 (8)	−0.0027 (5)	−0.0052 (6)	0.0112 (6)
C8	0.0319 (6)	0.0249 (6)	0.0491 (8)	0.0002 (5)	0.0068 (6)	0.0031 (6)
C9	0.0300 (6)	0.0275 (6)	0.0300 (6)	−0.0030 (5)	0.0043 (5)	−0.0008 (5)
C10	0.0234 (5)	0.0276 (6)	0.0260 (6)	−0.0037 (4)	−0.0022 (4)	−0.0004 (5)
C11	0.0292 (6)	0.0315 (7)	0.0258 (6)	−0.0018 (5)	0.0006 (5)	0.0007 (5)
C12	0.0333 (7)	0.0338 (7)	0.0369 (7)	−0.0064 (5)	−0.0017 (6)	0.0071 (6)
C13	0.0401 (8)	0.0297 (7)	0.0491 (9)	−0.0065 (6)	−0.0044 (6)	−0.0044 (6)
C14	0.0436 (8)	0.0439 (9)	0.0404 (8)	−0.0039 (7)	0.0045 (6)	−0.0158 (6)
C15	0.0331 (7)	0.0413 (8)	0.0306 (7)	−0.0064 (6)	0.0059 (5)	−0.0057 (6)
C16	0.0362 (7)	0.0374 (7)	0.0252 (6)	−0.0020 (6)	0.0002 (5)	0.0017 (5)
C17	0.0236 (5)	0.0349 (7)	0.0240 (6)	−0.0047 (5)	0.0021 (4)	0.0024 (5)
C18	0.0235 (6)	0.0380 (7)	0.0225 (6)	−0.0024 (5)	0.0041 (5)	0.0035 (5)
C19	0.0313 (6)	0.0379 (7)	0.0248 (6)	−0.0064 (5)	0.0009 (5)	0.0030 (5)
C20	0.0291 (6)	0.0480 (8)	0.0256 (6)	−0.0024 (6)	0.0001 (5)	0.0085 (6)
C21	0.0317 (7)	0.0482 (8)	0.0301 (7)	0.0097 (6)	0.0088 (5)	0.0077 (6)
C22	0.0386 (7)	0.0406 (8)	0.0321 (7)	0.0076 (6)	0.0068 (6)	−0.0027 (6)
C23	0.0317 (6)	0.0414 (8)	0.0235 (6)	0.0031 (5)	0.0009 (5)	−0.0024 (5)
C24	0.0609 (11)	0.0606 (11)	0.0427 (9)	0.0267 (9)	0.0043 (8)	0.0063 (8)

### Geometric parameters (Å, º)

**Table:**

S1—C1	1.7447 (13)	C19—C20	1.385 (2)
S1—C3	1.7559 (13)	C20—C21	1.383 (2)
N1—C2	1.4074 (16)	C21—C22	1.402 (2)
N1—C3	1.3823 (16)	C21—C24	1.505 (3)
N1—C10	1.4328 (17)	C22—C23	1.378 (2)
N2—N3	1.3983 (17)	C1—H1	0.9500
N2—C3	1.2894 (17)	C5—H5	0.9500
N3—C17	1.2946 (17)	C6—H6	0.9500
C1—C2	1.3381 (18)	C7—H7	0.9500
C2—C4	1.4762 (18)	C8—H8	0.9500
C4—C5	1.3938 (18)	C9—H9	0.9500
C4—C9	1.3986 (18)	C11—H11	0.9500
C5—C6	1.390 (2)	C12—H12	0.9500
C6—C7	1.383 (2)	C13—H13	0.9500
C7—C8	1.387 (2)	C14—H14	0.9500
C8—C9	1.384 (2)	C15—H15	0.9500
C10—C11	1.3834 (18)	C16—H16A	0.9800
C10—C15	1.3868 (19)	C16—H16B	0.9800
C11—C12	1.391 (2)	C16—H16C	0.9800
C12—C13	1.378 (2)	C19—H19	0.9500
C13—C14	1.391 (2)	C20—H20	0.9500
C14—C15	1.382 (2)	C22—H22	0.9500
C16—C17	1.4990 (19)	C23—H23	0.9500
C17—C18	1.4804 (19)	C24—H24A	0.9800
C18—C19	1.3996 (18)	C24—H24B	0.9800
C18—C23	1.399 (2)	C24—H24C	0.9800
			
C1—S1—C3	90.54 (6)	S1—C1—H1	124.00
C2—N1—C3	113.96 (10)	C2—C1—H1	124.00
C2—N1—C10	124.82 (11)	C4—C5—H5	120.00
C3—N1—C10	120.84 (10)	C6—C5—H5	120.00
N3—N2—C3	110.94 (11)	C5—C6—H6	120.00
N2—N3—C17	113.90 (11)	C7—C6—H6	120.00
S1—C1—C2	112.82 (10)	C6—C7—H7	120.00
N1—C2—C1	112.76 (11)	C8—C7—H7	120.00
N1—C2—C4	120.69 (11)	C7—C8—H8	120.00
C1—C2—C4	125.95 (11)	C9—C8—H8	120.00
S1—C3—N1	109.91 (9)	C4—C9—H9	120.00
S1—C3—N2	128.02 (10)	C8—C9—H9	120.00
N1—C3—N2	122.07 (12)	C10—C11—H11	120.00
C2—C4—C5	121.26 (11)	C12—C11—H11	120.00
C2—C4—C9	119.58 (11)	C11—C12—H12	120.00
C5—C4—C9	119.02 (12)	C13—C12—H12	120.00
C4—C5—C6	120.09 (12)	C12—C13—H13	120.00
C5—C6—C7	120.45 (13)	C14—C13—H13	120.00
C6—C7—C8	119.83 (13)	C13—C14—H14	120.00
C7—C8—C9	120.12 (13)	C15—C14—H14	120.00
C4—C9—C8	120.47 (12)	C10—C15—H15	120.00
N1—C10—C11	119.80 (11)	C14—C15—H15	120.00
N1—C10—C15	119.42 (12)	C17—C16—H16A	109.00
C11—C10—C15	120.78 (12)	C17—C16—H16B	109.00
C10—C11—C12	119.34 (12)	C17—C16—H16C	109.00
C11—C12—C13	120.31 (13)	H16A—C16—H16B	109.00
C12—C13—C14	119.87 (14)	H16A—C16—H16C	109.00
C13—C14—C15	120.31 (15)	H16B—C16—H16C	109.00
C10—C15—C14	119.35 (13)	C18—C19—H19	119.00
N3—C17—C16	124.23 (12)	C20—C19—H19	119.00
N3—C17—C18	116.36 (12)	C19—C20—H20	119.00
C16—C17—C18	119.37 (11)	C21—C20—H20	119.00
C17—C18—C19	121.26 (12)	C21—C22—H22	119.00
C17—C18—C23	121.15 (11)	C23—C22—H22	119.00
C19—C18—C23	117.54 (12)	C18—C23—H23	119.00
C18—C19—C20	121.03 (13)	C22—C23—H23	120.00
C19—C20—C21	121.36 (13)	C21—C24—H24A	109.00
C20—C21—C22	117.72 (14)	C21—C24—H24B	109.00
C20—C21—C24	120.83 (13)	C21—C24—H24C	109.00
C22—C21—C24	121.45 (15)	H24A—C24—H24B	110.00
C21—C22—C23	121.27 (14)	H24A—C24—H24C	109.00
C18—C23—C22	121.02 (12)	H24B—C24—H24C	109.00
			
C3—S1—C1—C2	−0.49 (11)	C2—C4—C9—C8	−174.46 (12)
C1—S1—C3—N1	−0.21 (9)	C5—C4—C9—C8	1.3 (2)
C1—S1—C3—N2	179.04 (13)	C4—C5—C6—C7	−1.0 (2)
C3—N1—C2—C1	−1.24 (15)	C5—C6—C7—C8	0.8 (2)
C3—N1—C2—C4	170.45 (11)	C6—C7—C8—C9	0.5 (2)
C10—N1—C2—C1	171.65 (11)	C7—C8—C9—C4	−1.6 (2)
C10—N1—C2—C4	−16.66 (18)	N1—C10—C11—C12	178.63 (12)
C2—N1—C3—S1	0.84 (13)	C15—C10—C11—C12	−1.2 (2)
C2—N1—C3—N2	−178.47 (12)	N1—C10—C15—C14	−177.75 (13)
C10—N1—C3—S1	−172.37 (9)	C11—C10—C15—C14	2.1 (2)
C10—N1—C3—N2	8.33 (18)	C10—C11—C12—C13	−0.6 (2)
C2—N1—C10—C11	−58.30 (17)	C11—C12—C13—C14	1.5 (2)
C2—N1—C10—C15	121.54 (14)	C12—C13—C14—C15	−0.6 (2)
C3—N1—C10—C11	114.13 (14)	C13—C14—C15—C10	−1.2 (2)
C3—N1—C10—C15	−66.03 (16)	N3—C17—C18—C19	168.14 (12)
C3—N2—N3—C17	−164.53 (12)	N3—C17—C18—C23	−9.15 (18)
N3—N2—C3—S1	2.76 (17)	C16—C17—C18—C19	−9.91 (18)
N3—N2—C3—N1	−178.07 (11)	C16—C17—C18—C23	172.80 (12)
N2—N3—C17—C16	2.60 (18)	C17—C18—C19—C20	−174.87 (12)
N2—N3—C17—C18	−175.34 (11)	C23—C18—C19—C20	2.51 (19)
S1—C1—C2—N1	1.06 (14)	C17—C18—C23—C22	175.55 (13)
S1—C1—C2—C4	−170.11 (10)	C19—C18—C23—C22	−1.8 (2)
N1—C2—C4—C5	136.04 (13)	C18—C19—C20—C21	−1.1 (2)
N1—C2—C4—C9	−48.28 (17)	C19—C20—C21—C22	−1.1 (2)
C1—C2—C4—C5	−53.44 (19)	C19—C20—C21—C24	178.69 (15)
C1—C2—C4—C9	122.24 (15)	C20—C21—C22—C23	1.8 (2)
C2—C4—C5—C6	175.69 (12)	C24—C21—C22—C23	−178.01 (15)
C9—C4—C5—C6	0.0 (2)	C21—C22—C23—C18	−0.3 (2)

### Hydrogen-bond geometry (Å, º)

Cg4 is the centroid of the C18–C23 ring.

**Table:**

*D*—H···*A*	*D*—H	H···*A*	*D*···*A*	*D*—H···*A*
C16—H16*B*···S1^i^	0.98	3.03	4.0031 (14)	175
C16—H16*C*···N2	0.98	2.28	2.7053 (18)	105
C15—H15···*Cg*4^i^	0.95	2.71	3.6170 (16)	160
C16—H16*A*···*Cg*4^ii^	0.98	2.77	3.5984 (16)	143

Symmetry codes: (i) −*x*+1, −*y*+1, −*z*+1; (ii) −*x*+2, −*y*+1, −*z*+1.
